# Plasma Proteomic Profile of Dual Declines in Cognitive and Physical Performance

**DOI:** 10.1093/geroni/igaf122.1912

**Published:** 2025-12-31

**Authors:** Alan Rathbun, Chixiang Chen, Michelle Shardell

**Affiliations:** University of Maryland School of Medicine, Baltimore, Maryland, United States; University of Maryland School of Medicine, Baltimore, Maryland, United States; University of Maryland School of Medicine, Baltimore, Maryland, United States

## Abstract

Dual declines in cognitive (or memory) and physical performance multiplicatively increase dementia risk, yet shared biological mechanisms remain unclear. This study investigated plasma proteins and biological mechanisms underlying dual cognitive-physical decline in older adults. Men and women (n = 774, age ≥ 60 years) enrolled in the Invecchiare in Chianti study were classified into three phenotypes—no decline, physical decline only, and dual decline—using group-based trajectory modeling of up to 15 years of cognitive or memory function (Mini-Mental State Examination) and 4-meter gait speed. Proteins were measured using aptamer-based proteomics (SomaLogic). Multinomial regressions, adjusted for potential confounders (age, sex, marital status, education, self-rated health, and body mass index), identified proteins that differed across phenotypes. Additionally, Reactome-based functional enrichment analysis highlighted pathways associated with dual decline. Eight proteins (q-value < 0.05) predicted dual cognitive and physical declines, five of which also predicted memory and physical declines. Elevated PI3, GDF15, TFF3, CCL15, TNNT2, and AGRP were associated with dual decline, while higher CKM and GHR were linked to no decline. Proteins differentiating dual decline from no decline were enriched in pathways regulating insulin-like growth factor (IGF) transport, IGF binding, and post-translational phosphorylation. Findings support a multi-system model in which inflammatory, metabolic, and muscle-related disruptions in IGF regulation and protein phosphorylation contribute to key hallmarks of aging. These findings can guide geroscience-driven interventions to prevent dual cognitive-physical decline and reduce dementia risk.

